# DNA damage-induced phosphatase Wip1 in regulation of hematopoiesis, immune system and inflammation

**DOI:** 10.1038/cddiscovery.2017.18

**Published:** 2017-04-03

**Authors:** B Uyanik, B B Grigorash, A R Goloudina, O N Demidov

**Affiliations:** 1 INSERM U866, University of Burgundy, Dijon, France; 2 Institute of Cytology RAS, St. Petersburg, Russia

## Abstract

PP2C serine–threonine phosphatase, Wip1, is an important regulator of stress response. Wip1 controls a number of critical cellular functions: proliferation, cell cycle arrest, senescence and programmed cell death, apoptosis or autophagy. *Ppm1d*, the gene encoding Wip1 phosphatase, is expressed in hematopoietic progenitors, stem cells, neutrophils, macrophages B and T lymphocytes in bone marrow and peripheral blood. The Wip1−/− mice display immunodeficiency, abnormal lymphoid histopathology in thymus and spleen, defects in B- and T-cell differentiation, as well as susceptibility to viral infection. At the same time, Wip1 knockout mice exhibit pro-inflammatory phenotype in skin and intestine in the model of inflammatory bowel disease (IBD) with elevated levels of inflammation-promoting cytokines TNF-*α*, IL-6, IL-12, IL-17. Several Wip1 downstream targets can mediate Wip1 effects on hematopoietic system including, p53, ATM, p38MAPK kinase, NF*k*B, mTOR. Here, we summarized the current knowledge on the role of Wip1 in the differentiation of various hematopoietic lineages and how Wip1 deficiency affects the functions of immune cells.

## Key points

- Wip1 phosphatase is a key negative regulator of DNA damage response and p53 signaling pathway.
- Wip1 is essential for normal development and differentiation of hematopoietic cells.
- Wip1 deficiency in immune system induces pro-inflammatory environment.

## Introduction

Wip1 phosphatase is a member of PP2C family of serine–threonine phosphatases.

Initially, it was cloned as p53 target gene and described as an important regulator of DNA damage response and tumorigenesis.^1^ Several proteins in the DNA damage signaling pathway are negatively regulated by Wip1 phosphatase. It was shown that Wip1 was able to dephosphorylate the DNA damage-induced phospho sites in histone H2AX, ATM and Chk2 kinases.^2,3^ Dephosphorylation of these proteins led to decreased signaling and reduced activation of p53, a transcriptional factor that turns ON expression of multiple effector genes in DNA repair, cell cycle, cell death and other programs involved in response to various stressors.^4–6^

In addition, Wip1 phosphatase is regulating the MAPK kinase pathway by dephosphorylating p38MAPK kinase^7,8^ and NF*κ*B.^9^

It was reasonable to assume that the wide spectrum of Wip1 interactome would not limit Wip1 functions only to DNA damage response and tumorigenesis. It was hypothesized that Wip1 may also be involved in other processes, including regulation of hematopoiesis. In fact, several of reported Wip1 targets including ATM, p53 and p38MAPK have an essential role in hematopoietic lineage differentiation.^10–12^ In line with this, we and others recently published several reports highlighting the role of Wip1 in myeloid and lymphocyte differentiation.^11,13,14^ The aim of this review is to describe our current knowledge on the role of Wip1 in hematopoiesis and immune system.

## Wip1 in T-cell differentiation

As was mentioned earlier, the role of Wip1 in the regulation of DNA damage response is well established. Wip1 inhibition or genetic deletion leads to hypersensitivity of p53 to upstream signaling and, as a result, augmented cell cycle arrest or apoptosis.

Certain stages of T-cell differentiation are characterized by increased DNA damage due to re-arrangement of T-cell receptor (TCR).^15^ Obviously, absence of the negative regulator, Wip1, during these stages may lead to perturbations in the T-cell development.

Using Wip1-deficient mice, Choi *et al.*^16^ showed an absence of proliferative response of T cells in Wip1−/− mice. Moreover, the authors observed abnormal lymphoid organs' histopathology and susceptibility to viral infection. This phenomenon was explained by Schito *et al.,*^13^ who demonstrated the importance of Wip1 in maturation of T cells in the thymus.^13^ T-cell development is controlled by well-determined spatial and temporal organization that starts with the entry in the thymus of double-negative (DN, CD4−CD8−) lymphoid progenitors, which subsequently progress through four stages of development (DN1 to DN4) to become double-positive (DP, CD4+CD8+), to undergo a re-arrangement of their TCR and then a positive or negative selection during the transition to single-positive T lymphocytes.^17^

Schito *et al.*^13^ determined that the block of T-cell development at the DN3 stage led to a reduced number of DP thymocytes. They were prone to apoptosis, subject to abnormal cell cycle and associated with reduced size of lymphoid organs such as thymus. Previously, Diehl *et al.*^18^ showed in 2000 that the inhibition of p38MAPK inside the thymus is required to allow the development of T cell to the DN4 stage. However, Schito *et al.* observed that p38MAPK inhibition could not rescue normal phenotype of T-cell development in Wip1−/− mice. In turn, the T-cell defect could be rescued by simultaneous deletion of p53, another downstream target of Wip1 phosphatase. In addition, we observed normalization of thymus size in Wip1−/− and p53−/− double-knockout mice. Of note, this organ was significantly smaller in Wip1-deficient mice compared with control mice.^13^ Overall, these facts indicate that Wip1 controls cell death and cell cycle arrest at DN3 stage of T-cell development in a p53-dependent manner.

**Figure 1:**
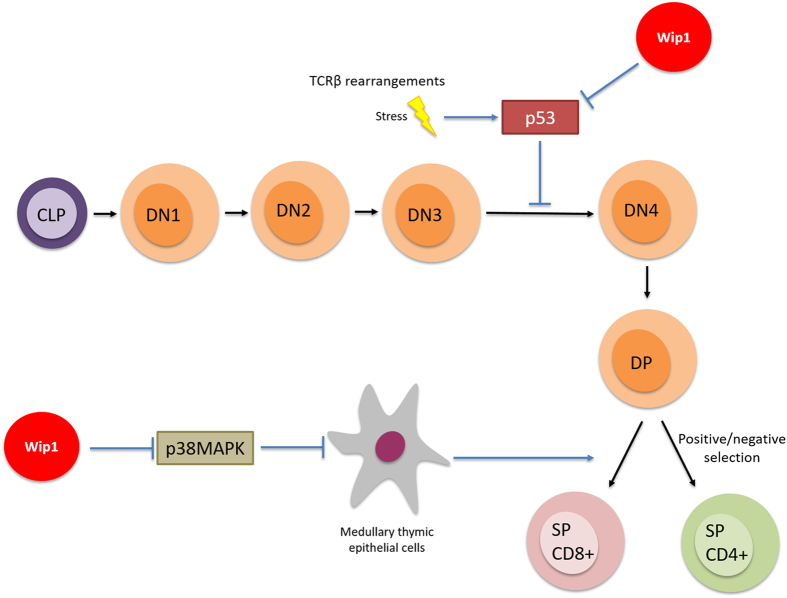
Wip1 in the regulation of T-cell differentiation.

Notably, a few years later, Sun *et al.*^19^ reported that Wip1 was a critical regulator of the functional thymic stroma. Indeed, thymic microenvironment is essential for the proper development of T cells. In line with this, Sun *et al.* observed defects in the differentiation and homeostasis of medullary thymic epithelial cells in Wip1-deficient mice. The authors confirmed our previous results, showing that Wip1-deficient mice had smaller thymic medulla in comparison to wild-type mice. They also concluded that Wip1 controls medullary thymic epithelial cell maturation in an intrinsic manner through negative regulation of the p38MAPK pathway. A plausible mechanism of Wip1 involvement in T-cell differentiation is shown in Figure 1.

Therefore, Wip1 allows the normal development of T lymphocytes and the maintenance of the functional organization of the thymus as a key organ of the immune system by preventing the hyperactivation of the p53 and p38MAPK pathways in thymic cells during maturation.

## Wip1 is a positive regulator of early B-cell development

Similarly to T-cell differentiation, differentiation of common lymphoid progenitor to B cell is known to be associated with mechanisms that generate elevated DNA damage pressure and p53 activation at a certain stage of development. Indeed, V(D)J recombination involved in B-cell receptor formation and subsequent B-cell maturation is initiated by DNA cleavage mediated by Rag1/2 endonucleases^20,21^ and repaired by the non-homologous DNA ends joining step pathway, thereby activating p53.^22^ Wip1 is a key regulator of DNA damage response and p53 homeostasis. Predictably, Wip1 deficiency has the same negative effect on B-cell development as it has on T-cell maturation.^13^

**Figure 2:**
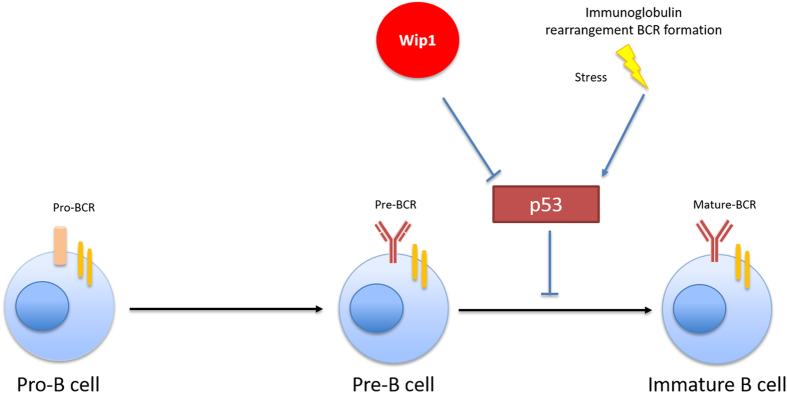
Wip1 in regulation of B-cell differentiation.

In this context, Yi *et al.*^14^ observed that Wip1-deficient mice possessed an abnormally low number of B cells not only in bone marrow (BM) but also in the spleen and peripheral blood. Loss of Wip1 functions was accompanied by severe disturbance at early stages of B-cell development with high mortality of B cells and a blockade of B-cell development at the pre-B stage. Similar to T-cell deficiency, B-cell deficiency in Wip1 knockout mice was rescued by a synchronic deletion of p53, showing that Wip1 is a guardian of B-cell maturation and proliferation by keeping in check the p53-mediated pro-apoptotic pathway, as shown in Figure 2.

## Wip1 controls differentiation of myeloid lineages

In sharp contrast to its effects in B- and T-cell development, Wip1 deficiency has positive effect on granulocyte differentiation. Indeed, it has been shown that Wip1−/− mice exhibited an important differentiation of myeloid lineages toward the granulocytic population, along with severe neutrophilia.^23^ Wip1 expression was progressively enhanced inside neutrophils during maturation of HSC, whereas its expression at early stages of development in progenitors was low. Under normal conditions, the increase in Wip1 expression during maturation and production of neutrophils prevent the differentiation of common myeloid progenitors (CMPs) to pro-inflammatory mature granulocytes to the detriment of other myeloid lineages. The authors have shown that Wip1 controls the development and maturation of neutrophils by having an inhibitory effect on the p38MAPK-STAT1 pathway. p38MAPK kinase is known to induce activation of transcription factors such as STAT1.^24,25^ Consequently, STAT1 turns ON expression of the CEBP/*α* protein, which mediates the differentiation of CMP to mature granulocytes. Unfortunately, to confirm the involvement of p38MAPK and STAT1 in manifestation of the Wip1-deficient phenotype, the authors used only chemical inhibitors of p38MAPK and STAT1 that can display nonspecific effects. The confirmation to the proposed mechanism would be a genetic deletion of p38MAPK and/or STAT1, which awaits future experiments similar to the genetic deletion of Wip1 in experiments with B- and T-cell lineages.

It is important to note that contrary to the p53-dependent phenotype of Wip1-deficient B- and T cells, p53 deletion did not rescue neutrophilia in Wip1−/− mice.

**Figure 3:**
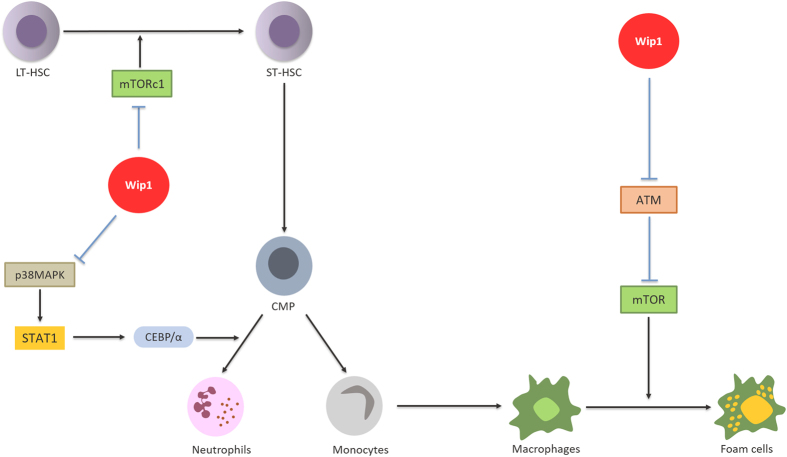
Wip1 in regulation of hematopoietic stem cells and myeloid-lineage differentiation.

Currently, little is known about the role of Wip1 in functioning of macrophages. The only indication of the importance of Wip1 in macrophages was mentioned with respect to the mouse model of atherosclerosis. Wip1 deletion prevented the formation of atherosclerotic plaques by suppression of the macrophages' conversion into foam cells.^26^ The authors observed the ATM-dependent inhibition of the mammalian target of rapamycin (mTOR) pathway in Wip1-deficient macrophages that was manifested by reduced phosphorylation of mTOR and downstream mTOR targets. Interestingly, the described mechanism did not involve such well-known targets of Wip1 as p53 and p38MAPK, and was triggered only by hyperactivation of ATM kinase in Wip1−/− mice. Figure 3 represents a general scheme of myeloid-progenitor differentiation and implication of Wip1.

## Wip1 is involved in regulation of HSC

In addition to the role of Wip1 in differentiating cells, lack of Wip1 can affect stem cell functions. It was shown previously that Wip1 is expressed and it has an essential role in the fate of intestinal stem cells and mesenchymal stem cells.^27,28^

Hematopoietic stem cells (HSC) are multipotent cells, able to give rise to both lymphoid and myeloid progenitor cells, subsequent differentiation of which provides all types of blood cells.^29^ HSCs consist of two populations: long-term HSC (LT-HSC) and short-term HSC (ST-HSC). LT-HSCs are ‘true’ stem cells that divide asymmetrically and produce an early progenitor cells, called ST-HSC. Wip1 was shown to be expressed at higher levels in LT-HSC than in ST-HSC that implicated Wip1 in the process of HSC differentiation and regulation of their activity.^30^ The premature aging phenotype of Wip1−/− mice correlates with behavior of aging HSCs, with the tendency to be more likely differentiated into myeloid progenitors.^31^ Further investigations shed a light on how Wip1 modulates the regenerative capacity of HSCs during hematopoietic injury. Being transplanted into the lethally irradiated host, Wip1−/− BM cells showed remarkable loss of regenerative capacity. Recipient mice, which received Wip1−/− cells, died in 4 weeks after the whole-body ionizing radiation treatment. Meanwhile, mice, which received wild-type BM cells, survived post treatment for at least 20 weeks.^30^ *In vitro* studies showed impaired ability of Wip1−/− LT-HSC and ST-HSC cells to form colonies of multipotent, erythroid and myeloid progenitors in methylcellulose-based colony-forming unit assay.^30^ As one of the major targets of Wip1, p53 was evaluated for the governing role in this process. The study of mice with double Wip1 and p53 deletion revealed the p53 dependence of Wip1-mediated differentiation of HSC. The authors concluded that Wip1 modulates differentiation and activity of HSC at early stages in cells with the highest pluripotency.^30^

Another side effect of aging HSCs is their expansion manifested by elevated levels of HSCs in BM and peripheral blood.^32^ Phenotype of Wip1−/− mice recapitulates this effect. Interestingly, despite the fact that many consequences of Wip1 deletion are linked to hyperactivation of the p53 pathway, Wip1-mediated accumulation of HSC is a p53-independent process. The global gene expression analysis indicated the upregulation of mTOR target genes in Wip1−/− HSC. It is well established that mTOR in the complexes with other proteins regulates cell growth, survival, protein synthesis and autophagy.^33,34^ mTOR is also involved in aging of HSC.^35^ Mice lacking one of the major downstream effectors of mTOR, ribosomal protein S6 kinase 1 (S6k1−/−), showed more quiescent phenotype of HSC, close to the one observed in mice with dietary restriction.^36^ These findings suggest that mTOR could serve as a regulator of HSC differentiation and expansion. The Brdu incorporation assay has shown that Wip1 inhibition stimulated expansion of HSC. The Wip1-dependent accumulation of HSC was completely blocked by the inhibition of mTOR with rapamycin. In contrast with the importance of mTOR in Wip1-dependent HSC self-proliferation, mTOR was dispensable for survival and differentiation of HSC. Rapamycin treatment of lethally irradiated mice with transplanted Wip1−/− BM cells did not rescue the phenotype. Interestingly, contrary to downregulation of mTOR in Wip1−/− macrophages, Wip1 deficiency has an opposite effect on the mTOR pathway in HSC.

Altogether, these data reveal the Wip1 role in modulation of the HSC functional activity and differentiation. In physiological conditions, Wip1 activity is dedicated to maintenance of HSC quiescence and facilitates their differentiation. Wip1 deficiency leads to premature aging of HSC, associated with higher self-proliferation rates and poorer differentiation, resulting in less regenerative capacity of Wip1−/− BM cells.

## Wip1 in regulation of immune response

In the first analysis of the phenotype of Wip1 knockout mice, Choi *et al.*^16^ reported the pro-inflammatory phenotype of Wip1−/− animals. The author observed that mice deficient for Wip1 exhibits a hyperplasia of lymphoid organs along with structural disorganization of these organs. Moreover, they also have shown that these mice were prone to chronic inflammation in normal tissue such as skin ulcerations and effects in immunocompetence with defects in T- and B-cell proliferative responses to mitogenic and antigenic stimuli.

Given the differential role of Wip1 in the development of hematopoietic lineages, the pro-inflammatory phenotype in Wip1−/− mice could be explained by hyperactivation of innate immunity. Sun *et al.*^37^ proposed that the lack of negative regulation by Wip1 can lead to sustained activation of the NF*κ*B pathway along with an abnormal production of pro-inflammatory cytokines by immune cells.

In this context, Hu and colleagues^38^ studied the pro-inflammatory behavior of Wip1-deficient neutrophils in mouse models using inflammatory bowel disease (IBD) models. They confirmed the previously obtained^9,37,39^ data and showed that Wip1−/− mice produced more pro-inflammatory cytokines such as TNF-*α*, IL-6, IL-12 and IL-17 than WT mice. The elevation of these cytokine can be linked to the ability of Wip1 to regulate p53 and NF*k*B pathways.^40,41^ These data suggest that Wip1 in immune cells intrinsically controls the sensitivity to DSS-induced colitis through regulation of cytokine production. Yet, the deficiency in Wip1 activity is associated with more severe inflammatory response. The authors used the full BM chimera models to show that Wip1-deficient neutrophils through enhanced secretion of IL-17 in colon increases the sensitivity to DSS-induced colitis. In sum, the Wip1 phosphatase is an intrinsic negative regulator of many pro-inflammatory cytokines and seems especially important for the control of pro-inflammatory behavior of neutrophils.

Our own data demonstrated that Wip1-deficient skin had more robust inflammatory response in the model of TPA-driven inflammation compared to skin of wild-type mice. The more severe inflammation in Wip1−/− skin was accompanied by a higher production of TNF-*α* and a number of other pro-inflammatory cytokines, but contrary to DSS-induced colitis it was not associated with accumulation of IL-17.^42^

## Conclusion

**Figure 4:**
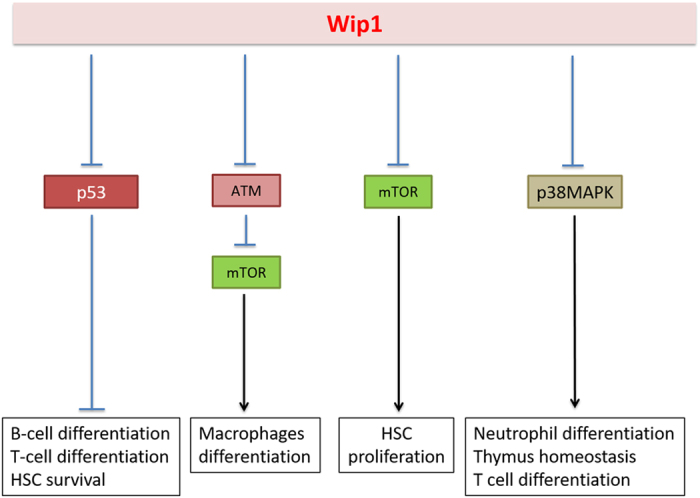
The summary of various effects of Wip1 on hematopoietic system.

In conclusion, we would like to point out that Wip1 deficiency severely affects the hematopoietic system and the properties of various hematopoietic lineages. Several established Wip1 targets, such as p53, p38MAPK kinase, ATM, NF*k*B mediate these effects (see Figure 4 for the details). It is important to emphasize that, despite obvious Wip1-dependent perturbations in differentiation of hematopoietic cells, the blood formula in young Wip1−/− mice does not differ from the blood formula of wild-type mice. The changes attributed to Wip1 could be observed only with age, resulting in neutrophilia and lymphopenia.
